# Interplay between ChREBP and SREBP-1c coordinates postprandial glycolysis and lipogenesis in livers of mice[S]

**DOI:** 10.1194/jlr.M081836

**Published:** 2018-01-15

**Authors:** Albert G. Linden, Shili Li, Hwa Y. Choi, Fei Fang, Masashi Fukasawa, Kosaku Uyeda, Robert E. Hammer, Jay D. Horton, Luke J. Engelking, Guosheng Liang

**Affiliations:** Departments of Molecular Genetics,* University of Texas Southwestern Medical Center, Dallas, TX 75390; Biochemistry,† University of Texas Southwestern Medical Center, Dallas, TX 75390; Internal Medicine,** University of Texas Southwestern Medical Center, Dallas, TX 75390; Veterans Affairs Medical Center,§ Dallas, TX 75216

**Keywords:** carbohydrate-responsive element-binding protein, sterol regulatory element-binding protein -1c, adeno-associated virus

## Abstract

Lipogenesis in liver is highest in the postprandial state; insulin activates SREBP-1c, which transcriptionally activates genes involved in FA synthesis, whereas glucose activates carbohydrate-responsive element-binding protein (ChREBP), which activates both glycolysis and FA synthesis. Whether SREBP-1c and ChREBP act independently of one another is unknown. Here, we characterized mice with liver-specific deletion of ChREBP (*L-Chrebp*^−/−^ mice). Hepatic ChREBP deficiency resulted in reduced mRNA levels of glycolytic and lipogenic enzymes, particularly in response to sucrose refeeding following fasting, a dietary regimen that elicits maximal lipogenesis. mRNA and protein levels of SREBP-1c, a master transcriptional regulator of lipogenesis, were also reduced in *L-Chrebp*^−/−^ livers. Adeno-associated virus-mediated restoration of nuclear SREBP-1c in *L-Chrebp*^−/−^ mice normalized expression of a subset of lipogenic genes, while not affecting glycolytic genes. Conversely, ChREBP overexpression alone failed to support expression of lipogenic genes in the livers of mice lacking active SREBPs as a result of Scap deficiency. Together, these data show that SREBP-1c and ChREBP are both required for coordinated induction of glycolytic and lipogenic mRNAs. Whereas SREBP-1c mediates insulin’s induction of lipogenic genes, ChREBP mediates glucose’s induction of both glycolytic and lipogenic genes. These overlapping, but distinct, actions ensure that the liver synthesizes FAs only when insulin and carbohydrates are both present.

The liver plays a central role in coordinating carbohydrate and lipid metabolism. During fasting, the liver maintains blood glucose by breaking down glycogen (glycogenolysis) and by producing new glucose (gluconeogenesis). Upon feeding, the liver converts ingested glucose into glycogen (glycogenesis) and pyruvate (glycolysis). Glycogen serves as a short-term energy supply in liver and muscle. Pyruvate can enter the mitochondria and be converted into acetyl-CoA, which can enter the Krebs cycle to supply NADH for oxidative phosphorylation, or to produce citrate for FA synthesis. De novo lipogenesis, the pathway of converting acetyl-CoA into FAs, is highly regulated at multiple levels (1). Activities of key glycolytic and lipogenic enzymes are acutely regulated by posttranslational modifications and allosteric mechanisms. The long-term response to excess carbohydrate is the transcriptional activation of glycolytic and lipogenic genes, controlled by complex regulatory processes that require the concerted actions of both insulin and glucose (1, 2). The two major transcription factors responsible for the coordinated induction of glycolytic and lipogenic genes are SREBP-1c and carbohydrate-responsive element-binding protein (ChREBP) (3–10).

Insulin’s stimulation of lipogenic gene expression is mediated by SREBP-1c, a transcription factor that activates all of the genes required for FA synthesis and the first enzyme in triglyceride (TG) synthesis (8). SREBP-1c mediates this activation through sterol regulatory element (SRE) motifs in the regulatory regions of lipogenic genes (11, 12). SREBP-1c, like the other two SREBP isoforms (SREBP-1a and SREBP-2), is synthesized as an inactive membrane-bound protein in the endoplasmic reticulum. To generate the active nuclear form, SREBP-1c needs to be transported by Scap to the Golgi, where it is processed sequentially by two proteases that release the active N-terminal fragment for entry into the nucleus (12–14).

The expression of SREBP-1c is highest in the liver, and it is the only SREBP isoform induced by insulin (15, 16). Insulin activates the transcription of SREBP-1c mRNA as well as the proteolytic processing of SREBP-1c precursor (17–19). The essential role of SREBP-1c in the insulin-stimulated increase of lipogenic gene expression was demonstrated in mice with genetic deletion of *Srebp-1c* or *Scap*. In these experiments, animals are fasted to provoke hunger and then fed or “refed” a high-carbohydrate/fat-free diet, so that postprandial physiological changes are accentuated (20). Deletion of *Srebp-1c* alone diminished (by 50%) refeeding-induced increases in levels of hepatic lipogenic mRNAs and rates of FA synthesis (20). The partial refeeding response in livers of *Srebp-1c* knockouts was due to a compensatory upregulation of SREBP-2, which can partially activate the lipogenic mRNAs in the absence of SREBP-1c (20). When the hepatic activity of all three SREBPs was blocked as a result of *Scap* deficiency (*L-Scap*^−/−^), the lipogenic response to refeeding was completely abolished (20–22).

SREBP-1c alone, however, is not sufficient to account for the synergistic induction of glycolytic and lipogenic genes in response to both insulin and glucose (2, 23, 24). Glucose activates many of these genes through the carbohydrate-responsive element (ChoRE), which is physically distinct from the insulin-responsive SRE motif (25–27). The glucose-sensing transcription factor that binds ChoRE is ChREBP, also a member of basic helix-loop-helix leucine zipper (bHLH-Zip) transcription factor family (28). ChREBP mRNA is broadly expressed, with the highest expression in metabolically active tissues, such as liver, adipose, brain, intestine, kidney, and pancreatic islets (28–31). Its major isoforms, ChREBP-α and the recently identified ChREBP-β, arise from the use of alternative promoters (32). The canonical ChREBP-α isoform encodes an 864-amino acid protein containing several functional domains, including a glucose-sensing domain, proline-rich region, and the DNA-binding bHLH-Zip motif. Glucose activates ChREBP-α through multiple mechanisms by inducing nuclear translocation, protein-protein interactions, and transcriptional activity (3, 4, 7, 9, 33). ChREBP-β, which lacks most of the N-terminal glucose-sensing domain, is constitutively active and requires feed-forward glucose-mediated activation of ChREBP-α to induce its expression (32). Other monosaccharides, particularly fructose, also stimulate ChREBP, although the mechanisms are not completely understood (7, 9, 34, 35).

The essential role of ChREBP in glucose and lipid metabolism has been demonstrated in vivo in rodents in which *Chrebp* was ablated by gene deletion (29) or knocked down by adenovirus-mediated RNA interference (36) or antisense oligonucleotides (37). Overall, these studies demonstrated that ChREBP is required for the increase of glycolytic and lipogenic mRNAs in response to excess carbohydrates, especially fructose. The latter role of ChREBP, in regulating fructose metabolism, was discovered in mice with germline deletion of *Chrebp* (*Chrebp*^−/−^ mice) (29). Although *Chrebp*^−/−^ mice consumed normal amounts of a standard chow diet, which contains starch as the primary carbohydrate, they would not ingest a high-sucrose or high-fructose diet and became moribund within a few days (29). The inability of *Chrebp*^−/−^ mice to tolerate sucrose precluded further studies exploring the in vivo role of ChREBP in the hepatic response to refeeding a high-sucrose diet after fasting, a condition in which glycolytic and lipogenic genes are maximally induced (1). Another confounding factor in studies of liver metabolism in *Chrebp*^−/−^ mice was the widespread expression of ChREBP in extrahepatic tissues (28–31). The hepatic contribution of ChREBP, therefore, could not be convincingly defined in studies of *Chrebp*^−/−^ mice.

To circumvent these limitations of *Chrebp*^−/−^ mice, we have produced and characterized a line of mice with liver-specific ChREBP deficiency (*L-Chrebp*^−/−^ mice). In marked contrast to the fructose-intolerant *Chrebp*^−/−^ mice, the *L-Chrebp*^−/−^ mice consume and tolerate fructose-containing diets, suggesting that hepatic ChREBP deficiency does not contribute to dietary fructose intolerance. The creation of these mice also permitted the study of the interdependent roles of SREBP-1c and ChREBP in regulating the expression of glycolytic and lipogenic mRNAs in liver under basal and carbohydrate-induced states. Combined, our data show that both SREBP-1c and ChREBP are required for the maximal induction of postprandial lipogenesis with fructose feeding. These studies further define the molecular events that contribute to the development of hepatic steatosis associated with excess dietary fructose consumption, which has become epidemic in Westernized societies (7, 35).

## MATERIALS AND METHODS

### Generation of liver-specific *Chrebp* knockout mice

A conditional targeting vector was produced by the insertion of a *loxP* site 150 bp upstream of exon 9 and a *loxP, frt*-flanked *pgkneopA* cassette (38) immediately downstream of exon 15. LR-2 ES cells, derived from albino C57BL/6N blastocysts, were cultured on leukemia inhibitory factor-producing STO feeder cells and transfected with the linearized targeting vector as described previously (20). Three positive ES clones were expanded and injected into C57BL/6J blastocysts to obtain chimeric males. All resulting chimeric males were used to cross to C57BL/6N females to obtain offspring that carried the *floxed Chrebp* allele with the *loxP, frt*-flanked *pgkneopA* cassette (*Chrebp*^+/^*^fneo^*). Because the presence of the *pgkneopA* cassette causes inactivation of *Chrebp* (data not shown), we bred the *Chrebp*^+/^*^fneo^* mice with a strain of transgenic mice that express a *Flp1* recombinase gene under the direction of the human *ACTB* promoter (Jackson Laboratory; #003800) to remove the *frt*-flanked *pgkneopA* cassette. The resulting *Chrebp*^+/^*^f^* mice were then intercrossed to produce mice homozygous for the *floxed* allele without the selection cassette (*Chrebp ^f/f^*). *Chrebp ^f/f^* mice were bred to *Albumin-Cre* transgenic mice (Jackson Laboratory; 003574) to derive mice homozygous for the *floxe*d *Chrebp* allele and hemizygous for the *Alb*-Cre transgene (*Chrebp^f/f^;Alb-Cre*). For brevity, we designate the *Chrebp^f/f^;Alb-Cre* mice as *L-Chrebp*^−/−^ (liver-specific *Chrebp* knockout). Littermate *Chrebp^f/f^* mice were used as controls for all of the experiments. To genotype mice, ear-punch DNA was prepared with a direct lysis kit (Viagen Biotech Inc.) and used for PCR with the primers, 5′-GAAAGGGGTTGGGATCCAAGGGTCC-3′ and 5′-GTGGCTGAGTGGATCATCTGTAAGACTGAT-3′. Ear-punch DNA of wild-type and *floxed* alleles produced PCR products of 300 and 350 bp, respectively.

### Animal studies and diets

All animal experiments described in this work were approved and conducted under the oversight of the University of Texas Southwestern Institutional Animal Care and Use Committee. All mice were housed in colony cages in a room with a 12 h light/12 h dark cycle and were fed a standard chow diet (Teklad Global Rodent Diet 2018). A high-sucrose diet with 60% (w/w) sucrose, 20% (w/w) casein protein, and 0% fat was purchased from MP Biomedicals (960238). For the fasting and refeeding experiments, mice were divided into three groups: nonfasted (N), fasted (F), and refed (R). The nonfasted group was fed the chow diet ad libitum, the fasted group was fasted for 12 h, and the refed group was fasted for 12 h and then refed with the high-sucrose diet for 12 h prior to study. The starting times for the fasting and refeeding experiments were staggered so that all mice were euthanized at the same time, which was at the end of dark cycle.

### Metabolic parameters

Animals were euthanized with isoflurane, blood was obtained from the inferior vena cava in EDTA-coated tubes, and plasma was separated and stored at −80°C. Glycogen content of liver was measured with an assay kit from Biovision (K646-100). Plasma glucose, aspartate transaminase, and alanine transaminase concentrations were measured using the Vitros 250 chemistry analyzer (Ortho Clinical Diagnostics). Plasma insulin concentration was measured with an ELISA kit from Crystal Chem, Inc. (90080).

### Immunoblot analysis

Liver whole-cell lysates and membrane fractions were prepared individually and equal amounts of protein from each mouse of the same group were pooled (38, 39). Aliquots of pooled proteins were subjected to SDS-PAGE and immunoblot analysis. The primary antibodies were diluted in 5% BSA (A7906; Sigma-Aldrich) in PBS-0.05% Tween-20 (P3563; Sigma-Aldrich) and used at the indicated concentrations: ChREBP (Novus Biologicals; NB400-135, 1:1,000), SREBP-1 (IgG-20B12, 5 μg/ml) (40), SREBP-2 (IgG-22D5, 5 μg/ml) (40, 41), Scap (R139, 5 μg/ml) (21), Insig-1 (IgG, 5 μg/ml) (38, 42), Insig-2 (IgG, 5 μg/ml) (38, 42), green fluorescent protein (GFP) (Novus Biologicals; NB600-308, 1:5,000), and calnexin (Novus Biologicals; NB100-1974, 1:5,000). Rabbit monoclonal anti-SREBP-1 (IgG-20B12) was generated by first immunizing rabbits with a bacterially produced (His)_10_-tagged protein containing amino acids 33-250 of mouse SREBP-1a. B-cells from an anti-SREBP-1 antibody-producing rabbit were then fused with the rabbit hybridoma fusion partner, 240E-1, to obtain hybridoma clone 20B12, as described (43). Bound antibodies were visualized with peroxidase-conjugated affinity-purified secondary antibodies (Jackson Immuno Research) and the SuperSignal CL-HRP substrate system (Pierce). Filters were exposed to Kodak X-Omat™ Blue XB-1 film at room temperature for 1–30 s or imaged with a LI-COR Odyssey^®^ Fc dual-mode imaging system for quantification with LI-COR Image Studio™ software.

### Real-time RT-PCR

**Fig. 1.**
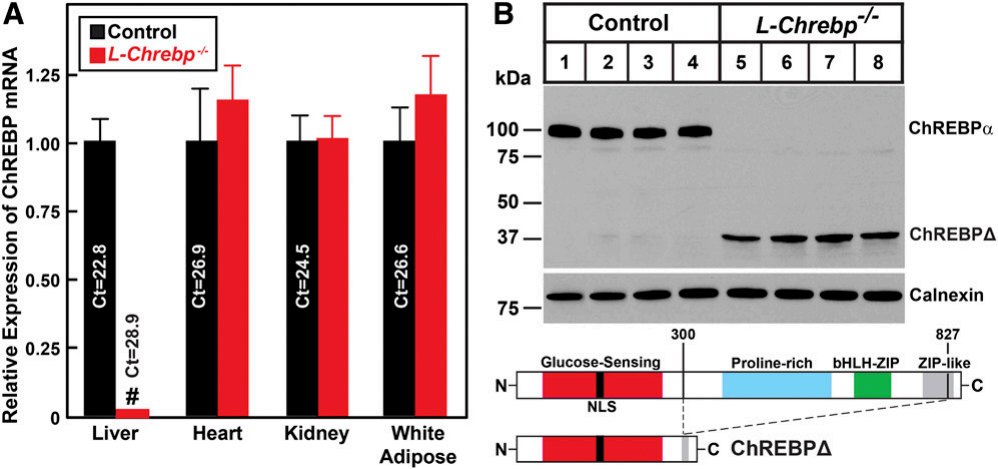
Liver-specific disruption of *Chrebp* in mice. A: Quantitative real-time PCR analysis of ChREBP mRNA. Total RNA was isolated from various tissues of control and liver-specific *Chrebp* knockout (*L-Chrebp*^−/−^) mice and subjected to real-time PCR analysis with 36B4 as the invariant control. Each value represents the mean ± SEM of four mice relative to that of controls, which was arbitrarily defined as 1.0. ^#^*P* < 0.01 denotes the level of statistical significance (two-tailed Student’s *t*-test) between control and *L-Chrebp*^−/−^ mice. B: Immunoblot analysis of ChREBP in liver lysates of control and *L-Chrebp*^−/−^ mice. Aliquots (60 μg of protein) of liver whole-cell lysates were subjected to SDS-PAGE and immunoblot analysis with anti-ChREBP and anti-calnexin antibodies. ChREBPΔ denotes a truncated aberrant ChREBP protein present only in lysates prepared from *L-Chrebp*^−/−^ livers. The functional domains of ChREBP, including the glucose-sensing proline-rich bHLH-Zip DNA-binding and ZIP-like domains, are denoted. NLS, nuclear localization sequence.

Total RNA was prepared from mouse tissues using an RNA STAT-60 kit from TEL-TEST “B” (Friendswood, TX) and subjected to real-time RT-PCR as previously described (20). All reactions were performed in triplicate and the relative amount of all mRNAs was calculated using the Comparative C_T_ method. Mouse 36B4 (Fig. 1A) or ApoB (all other studies) mRNAs were used as the invariant controls. Sequences of real-time PCR primers are listed in supplemental Table S1.

### Cloning of the aberrant ChREBPΔ transcript in *L-Chrebp*^−/−^ livers

Total RNAs isolated from *L-Chrebp*^−/−^ livers were used to synthesize first-strand cDNAs using the PrimeScript First Strand cDNA synthesis kit from Clontech (6110A), which were used as templates for PCR amplifications. Two PCR reactions were carried out with PfuUltra II polymerase (Agilent Technologies) using 5′ primers corresponding to the 5′ end of either ChREBP-α (5′-GTGGCCATGGCGCGCGCGCTGGCGGATC-3′) or ChREBP-β (5′-GACGCCATCTGCAGATCGCGTGGAGC-3′). Both reactions used the same 3′ primer, 5′-AGGATTATAATGGTCTCCCCAG­GGTGCC-3′, corresponding to the common 3′ end of ChREBP-α and ChREBP-β. The abundance of the PCR product amplified by ChREBP-α primers was >10-fold higher than that of ChREBP-β primers. Both products were cloned into pCR2.1-TOPO vector and sequenced. The results confirmed that the PCR product of ChREBP-α primers in the *L-Chrebp*^−/−^ livers encoded an aberrant ChREBP-α with an internal deletion of amino acid residues 301-826. Likewise, the PCR product of ChREBP-β primers encoded an aberrant ChREBP-β with the internal deletion of the same region. Because the aberrant ChREBP-β is expressed at an extremely low level in *L-Chrebp*^−/−^ liver (<3% of that of full-length ChREBP-β in control liver, see supplemental Tables S2A and S2B), only the aberrant ChREBP-α (designated as ChREBPΔ) was evaluated for activity.

### Recombinant adeno-associated virus

DNA fragments encoding ChREBPΔ or nuclear (n)SREBP-1c (amino acids 1-456) of mouse SREBP-1c were cloned into the pAAV-CAG-shuttle-WPRE vector (Applied Viromics), whose expression is under the control of cytomegalovirus enhancer and the chicken β-actin promoter. A DNA fragment encoding full-length mouse ChREBP-α was cloned into pAAV-TBG vector (Vector Biolabs), whose expression is under the control of the human thyroid hormone-binding globulin (TBG) promoter and microglobin/bikunin enhancer. The expression vectors were then packaged into liver-specific adeno-associated virus (AAV)-DJ (44) by large-scale transfection of HEK293 cells (fee-for-service at Vector Biolabs). Viral particles were purified by serial CsCl_2_ centrifugation and stored at 5% (v/v) glycerol in phosphate-buffered saline at −80°C. AAVs were administered by tail vein injection and the mice were studied 1–2 weeks after the injection, as indicated in the figure legends.

## RESULTS

### Liver-specific deletion of *Chrebp* by Cre-mediated recombination

Mice carrying the conditional *floxed Chrebp* allele (*Chrebp^f/f^*) were generated by inserting one *loxP* site upstream of exon 9 and another *loxP* site immediately downstream of exon 15 (supplemental Fig. S1). Cre-mediated recombination removed exons 9-15, which encode various functional domains of ChREBP, such as the proline-rich domain and the basic helix-loop-helix leucine zipper DNA-binding domain present in both ChREBP-α and ChREBP-β isoforms. To generate liver-specific deletion of ChREBP, *Chrebp^f/f^* mice were bred to transgenic mice that express Cre recombinase driven from the hepatocyte-specific albumin promoter (*Alb-Cre*) (45) to generate mice homozygous for the *floxed Chrebp* allele and hemizygous for the *Alb-Cre* transgene (*Chrebp^f/f^;Alb-Cre*). For brevity, we designate the liver-specific *Chrebp* knockout mice, *L-Chrebp*^−/−^. For all studies, we bred *Chrebp^f/f^;Alb-Cre* males with *Chrebp^f/f^* females to obtain control *Chrebp^f/f^* and *L-Chrebp*^−/−^ littermates. *L-Chrebp*^−/−^ mice were born at expected Mendelian ratios and were grossly indistinguishable from control littermates from birth to adulthood.

Real-time PCR was carried out to quantify the relative total ChREBP mRNA levels in the liver, heart, kidney, and white adipose tissues from control and *L-Chrebp*^−/−^ mice (**Fig. 1A**). Real-time PCR primers were designed to amplify a C-terminal region shared by ChREBP-α and ChREBP-β. Compared with control mice, the expression of ChREBP mRNA in *L-Chrebp*^−/−^ mice was reduced by 98% in liver. ChREBP mRNA levels in the other three tissues were comparable between control and *L-Chrebp*^−/−^ mice. Thus, the *Alb-Cre*-mediated recombination led to an efficient and hepatocyte-specific ablation of *floxed Chrebp* alleles.

Figure 1B shows immunoblot analysis of ChREBP using an antibody directed against a peptide antigen located in the C-terminal region common to both ChREBP-α and ChREBP-β. A protein band of 100 kDa, corresponding to the predicted full-length ChREBP-α protein, is present in control but absent in *L-Chrebp*^−/−^ livers. The predicted ChREBP-β protein band (75 kDa) was not detectable in control or *L-Chrebp*^−/−^ livers, likely due to instability of the ChREBP-β protein (32). A protein band of 38 kDa (denoted as ChREBPΔ) was detected in lysates prepared from *L-Chrebp*^−/−^ livers. To confirm the identity of this aberrant ChREBPΔ, we performed PCR with ChREBP primers on first strand cDNA synthesized from mRNA prepared from *L-Chrebp*^−/−^ livers. Subsequent cloning and sequencing verified that *L-Chrebp*^−/−^ livers, as a result of the Cre-mediated removal of exons 9-15, produce a truncated ChREBP-α transcript encoding a 38 kDa aberrant ChREBPΔ protein lacking amino acid residues 301-826. The ChREBPΔ protein, which does not contain the proline-rich and bHLH-Zip domains, would not be expected to bind to the ChoRE or to affect transcription. To confirm this hypothesis, we prepared a recombinant AAV encoding ChREBPΔ and administered AAV-ChREBPΔ to wild-type mice. As shown in supplemental Fig. S2, the AAV-mediated overexpression of ChREBPΔ did not alter the mRNA levels of ChREBP or its target genes, which suggests that ChREBPΔ is not a transcriptional activator and does not impair the transcriptional activity of endogenous ChREBP (i.e., act as a dominant-negative regulator).

### ChREBP deficiency in liver does not cause fructose intolerance

**Fig. 2.**
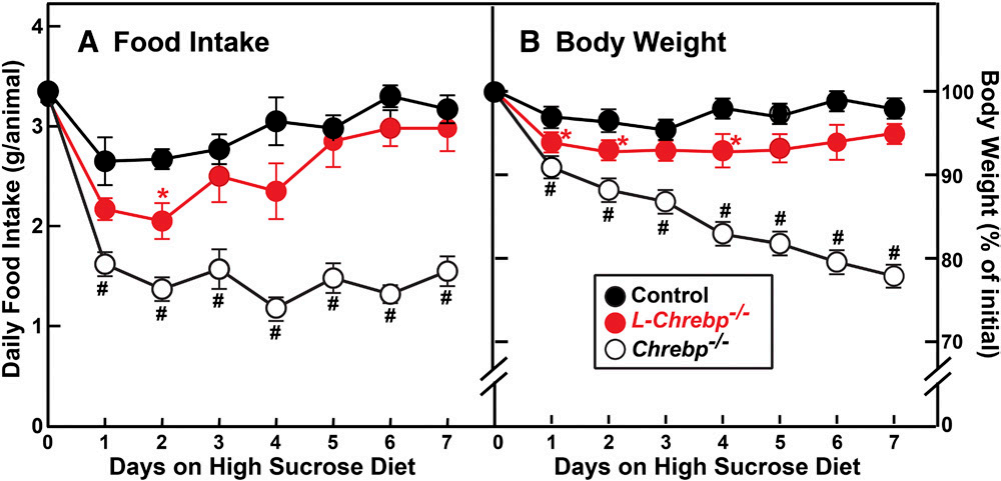
Effect of a high-sucrose diet on germline (*Chrebp*^−/−^) and liver-specific (*L-Chrebp*^−/−^) *Chrebp* knockout mice. Control, *Chrebp*^−/−^, and *L-Chrebp*^−/−^ mice (male, 2 months of age) were individually housed and fed ad libitum chow diet containing 44% (w/w) complex carbohydrates in the form of wheat and corn starch, and then switched to a 60% (w/w) high-sucrose diet for another 7 days. Food intake (A) and body weight (B) of each mouse were monitored daily. Each value represents the mean ± SEM of six mice. Day 0 values of food intake denote the average daily consumption of chow diet prior to sucrose feeding. For clarity, the initial body weight of each animal prior to sucrose feeding was defined as 100%. *P < 0.05 and ^#^*P* < 0.01 denote the level of statistical significance (two-tailed Student’s *t*-test) between control and *L-Chrebp*^−/−^ or control and *Chrebp*^−/−^ mice.

We noted previously that *Chrebp*^−/−^ mice, although they consume normal amounts of starch-containing chow diet, would not ingest high-sucrose/fructose diets and became moribund within a few days (29). It was not clear, however, whether intolerance of dietary fructose in *Chrebp*^−/−^ mice was caused by either the hepatic or extrahepatic effects of ChREBP deficiency. In **Fig. 2**, we address this question by comparing the response to sucrose feeding in germline *Chrebp*^−/−^ and liver-specific *L-Chrebp*^−/−^ mice. The mice were individually housed and fed a chow diet ad libitum [44% (w/w) complex carbohydrates in the form of starch], and then switched to a high-sucrose diet containing 60% (w/w) sucrose. As shown in Fig. 2A, the daily intake of chow diet was comparable between control (3.4 g/animal), *L-Chrebp*^−/−^ (3.3 g/animal), and *Chrebp*^−/−^ (3.3 g/animal) mice. However, the three groups of mice showed drastically different responses once they were switched to the 60% sucrose diet (high-sucrose diet). Consistent with our previous finding (29), the daily intake of high-sucrose diet (1.4 g/animal) by *Chrebp*^−/−^ mice was less than 50% of the daily intake of chow diet (3.3 g/animal) (Fig. 2A). As a result, over the course of 7 days of sucrose feeding, the *Chrebp*^−/−^ mice lost ∼20% of their initial body weights and became moribund (Fig. 2B). The control and *L-Chrebp*^−/−^ mice, on the other hand, adapted to the sucrose diet by day 5. Over days 5–7, the daily intake of high-sucrose diet by control (3.2 g/animal) and *L-Chrebp*^−/−^ (2.9 g/animal) mice was only slightly less than their daily intakes of chow diet (3.4 and 3.3 g, respectively). After 7 days of sucrose feeding, the control and *L-Chrebp*^−/−^ mice only lost 2% and 5% of their initial body weights, respectively (Fig. 2B). The data of Fig. 2 suggest that hepatic ChREBP deficiency alone does not lead to systemic tolerance of dietary sucrose. This finding is consistent with a recent study that showed that fructose tolerance requires intestinal, but not hepatic, ChREBP (46).

### ChREBP deficiency abolishes sucrose refeeding-induced increases of hepatic SREBP-1c glycolytic and lipogenic gene expression

The ability of the *L-Chrebp*^−/−^ mice to tolerate sucrose made it possible to explore the in vivo role of ChREBP in the hepatic response to refeeding of high-sucrose diet after fasting, a condition in which the glycolytic and lipogenic genes are maximally induced (5- to 20-fold above the normal fed state) (1). For this experiment, the control and *L-Chrebp*^−/−^ mice were fasted for 12 h (F) or fasted for 12 h and then refed for 12 h with a high-sucrose diet (R). Nonfasted (N) groups, which were fed chow diet ad libitum, are included as references. The phenotypic parameters and hepatic mRNA levels of the mice used in this experiment are summarized in supplemental Table S2A (nonfasted groups) and supplemental Table S2B (fasted, sucrose-refed groups). Plasma insulin concentrations were comparable in nonfasted control and *L-Chrebp*^−/−^ mice. Fasting and refeeding caused similar degrees of fall and rise of plasma insulin in both groups of mice. Plasma glucose concentrations were slightly lower in *L-Chrebp*^−/−^ mice. Relative to controls, the *L-Chrebp*^−/−^ mice had elevated liver glycogen content. Similar accumulations of hepatic glycogen were observed previously in whole-body *Chrebp*^−/−^ mice (29). Fasting reduced the glycogen levels in the livers of control and *L-Chrebp*^−/−^ mice to comparably low levels.

**Fig. 3.**
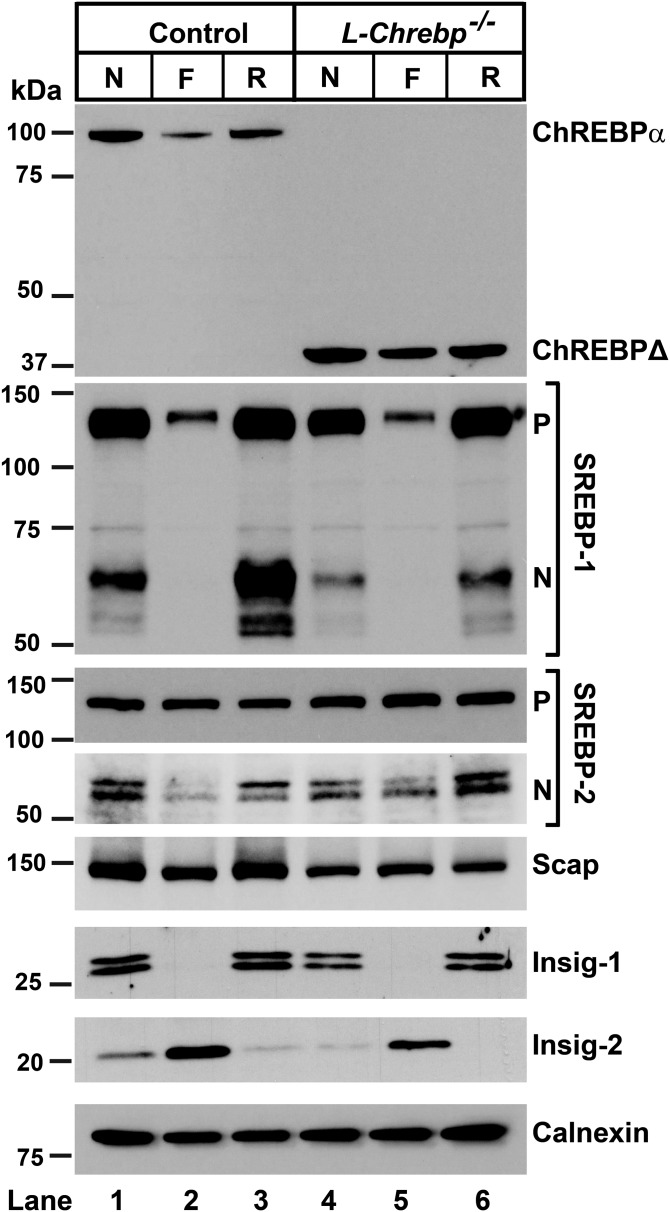
Immunoblot analysis of livers from control and *L-Chrebp*^−/−^ mice subjected to fasting and refeeding with a high-sucrose diet. Littermate control and *L-Chrebp*^−/−^ mice (same as those described in supplemental Tables S2A and S2B) were subjected to fasting and refeeding. The nonfasted (N) groups were fed chow diet ad libitum. The fasted (F) group was fasted 12 h, and the refed (R) group was fasted for 12 h and then refed with 60% (w/w) high-sucrose diet for 12 h prior to study. Liver whole-cell lysates and membrane fractions were prepared individually, and equal amounts of protein from each mouse of the same group (four per group) were pooled. Aliquots (40 μg for whole-cell lysates and 30 μg for membrane fractions) of the pooled protein were subjected to SDS-PAGE and immunoblot analysis. Immunoblot analysis of Insig-1 and Insig-2 were carried out using membrane fractions. Whole-cell lysates were used to detect other proteins. The precursor and nuclear forms of SREBPs are denoted as P and N, respectively. Calnexin was used as loading control.

In **Fig. 3**, protein levels of ChREBP and SREBPs were measured by immunoblot analysis in livers from control and *L-Chrebp*^−/−^ mice. The amount of ChREBP-α protein was reduced by fasting and restored upon refeeding in control mice (Fig. 3, lanes 1–3). The aberrant 38 kDa ChREBPΔ protein, but not the full-length 100 kDa ChREBP-α protein, was detected in the *L-Chrebp*^−/−^ livers (Fig. 3, lanes 4–6). The predicted 75 kDa ChREBP-β protein band was not detectable in any group, consistent with Fig. 1. As shown previously (20, 47), nSREBP-1 was reduced by fasting and induced to supranormal levels upon refeeding in control mouse livers. Relative to those of controls, livers from *L-Chrebp*^−/−^ mice exhibited reduced levels of nSREBP-1 protein in the nonfasted state (Fig. 3, lane 1 vs. lane 4). The “overshoot” of nSREBP-1 protein in the refed condition was also abolished in *L-Chrebp*^−/−^ mice (Fig. 3, lane 3 vs. lane 6). Levels of nSREBP-2 protein in *L-Chrebp*^−/−^ mice were similar (nonfasted) or slightly higher (fasted or refed) than those of controls under the matched dietary conditions. In both strains, Insig-1 fell during fasting and rose with refeeding, whereas Insig-2 was elevated by fasting and repressed by refeeding, as reported previously (42, 48). Relative to that of controls, the fasting-induced increase of hepatic Insig-2 was attenuated in *L-Chrebp*^−/−^ mice. The hepatic level of Scap protein in *L-Chrebp*^−/−^ mice was also slightly lower than controls, especially under refed conditions. Overall, the data suggests that the reduction in nSREBP-1 protein in *L-Chrebp*^−/−^ livers was not caused by defects in the general machinery of SREBP proteolysis. The slight increase of hepatic nSREBP-2 in *L-Chrebp*^−/−^ mice is likely a compensatory response to the lack of ChREBP and the associated decrease of nSREBP-1, as observed previously in *Srebp-1c*^−/−^ mice (20).

**Fig. 4.**
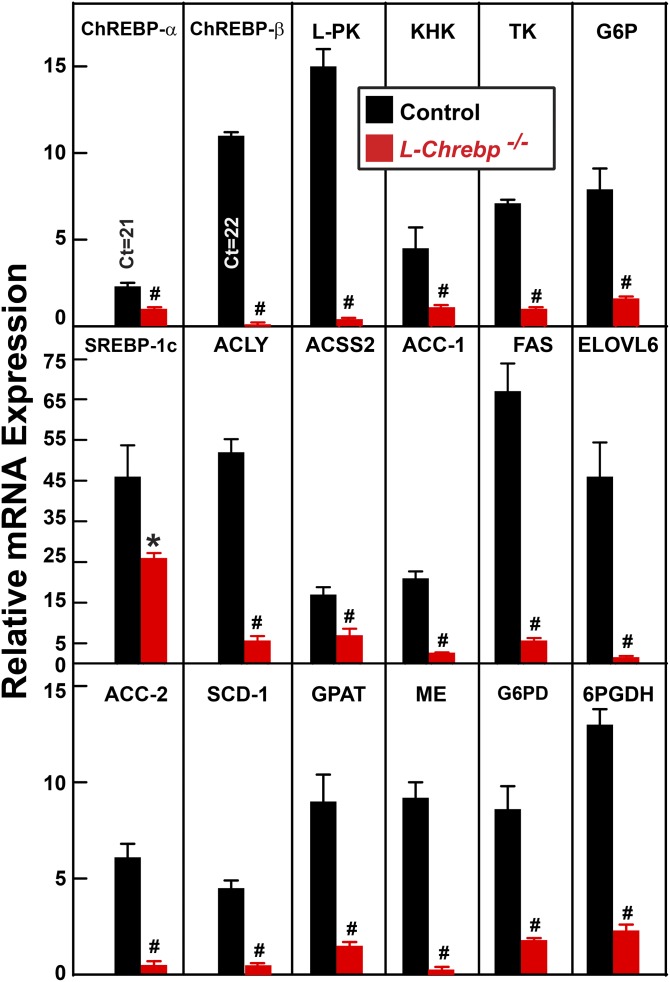
Relative amount of mRNAs encoding lipogenic and glycolytic enzymes in livers from sucrose-refed control and *L-Chrebp*^−/−^ mice. The mice used here were the same as those in Fig. 3. Total liver RNAs were prepared and subjected to real-time PCR analysis with ApoB as the invariant control. For brevity, only selected mRNAs from refed groups were shown here, and the mRNA expression was plotted as the amount relative to that in the livers of fasted control mice, which was arbitrarily defined as 1.0. Each bar represents the mean ± SEM of the values from four mice per group. *P < 0.05 and ^#^*P* < 0.01 denote the level of statistical significance (two-tailed Student’s *t*-test) between control and *L-Chrebp*^−/−^ mice. ChREBP-α and -β real-time PCR primers detect both wild-type (present in control livers) and aberrant (present only in in *L-Chrebp*^−/−^ livers) ChREBP-α and -β isoforms. The values for ChREBP-α and -β mRNAs in *L-Chrebp*^−/−^ livers reflect those of nonfunctional aberrant isoforms. Additional mRNA values from this experiment are shown in supplemental Tables S2A and S2B. ACSS2, acetyl-CoA synthetase 2; G6PDH, glucose-6-phosphate dehydrogenase.

**Figure 4** shows the relative mRNA levels of selected genes in the livers of sucrose-refed control and *L-Chrebp*^−/−^ mice. In refed controls, hepatic ChREBP-α mRNA was induced by a modest 2.3-fold (relative to the fasted value), whereas ChREBP-β mRNA was elevated 11-fold. The postprandial inductions of ChREBP-α and ChREBP-β mRNA are consistent with previous studies (32, 49, 50). In the *L-Chrebp*^−/−^ mice, only the aberrant ChREBP-α and -β mRNAs were detected. The level of the aberrant ChREBP-α mRNA (encoding ChREBPΔ protein) in *L-Chrebp*^−/−^ livers was approximately 40% that of full-length ChREBP-α mRNA in controls. ChREBP-β (full-length or aberrant) mRNA was nearly undetectable in *L-Chrebp*^−/−^ livers, consistent with the idea that ChREBP-β expression requires functional ChREBP-α (32).

In the livers of sucrose-refed control mice, mRNA levels of ChREBP-regulated genes encoding key enzymes of glucose metabolism were increased by 4.5- to 15-fold (relative to fasted values) (Fig. 4). These include ketohexokinase (KHK) (4.5-fold), triose kinase (TK) (7.1-fold), glucose-6-phosphatase (G6P) (7.9-fold), and L-pyruvate kinase (L-PK) (15-fold). The refeeding-induced increases of these mRNAs were abolished in *L-Chrebp*^−/−^ livers. Glucokinase (GK) mRNA, on the other hand, was elevated by ∼2-fold in *L-Chrebp*^−/−^ livers relative to that of controls (supplemental Tables S2A, S2B), similar to that in livers of germline *Chrebp*^−/−^ mice (29).

The level of SREBP-1c mRNA was increased by 46-fold (relative to fasted value) in sucrose-refed control mice (Fig. 4). The induction of SREBP-1c mRNA in *L-Chrebp*^−/−^ livers was 44% less than that measured in controls and partially explains the reduced SREBP-1 protein levels shown in Fig. 3. The mRNA levels of genes encoding enzymes of FA synthesis and NADPH production were also induced in the livers of control mice by refeeding, ranging from 4.5-fold for stearoyl-CoA desaturase 1 (SCD-1) to 67-fold for FAS. The refeeding-induced increases of mRNAs encoding these genes were all markedly blunted in *L-Chrebp*^−/−^ mice.

In contrast, mRNAs encoding cholesterol biosynthetic enzymes, which are predominantly controlled by SREBP-2, i.e., HMG-CoA synthase and HMG-CoA reductase, were induced to comparable levels in sucrose-refed control and *L-Chrebp*^−/−^ mice (supplemental Table S2B). These changes paralleled with levels of nSREBP-2 (Fig. 3), confirming that the reduction of nSREBP-1c in the *L-Chrebp*^−/−^ mice was not associated with a general blockade of SREBP processing or transcriptional activation. The mRNA levels of PPAR-α and PPAR-γ, two key transcriptional regulators of lipid and carbohydrate metabolism, were not different between control and *L-Chrebp*^−/−^ mice (supplemental Tables S2A, S2B).

Despite the dramatic decreases in lipogenic mRNAs, the hepatic TG contents in *L-Chrebp*^−/−^ mice were paradoxically higher than those of control mice under all three dietary conditions (supplemental Tables S2A, S2B). The increase of hepatic TG in the face of decreased hepatic lipogenesis was also observed in other studies of fructose-fed mice (46) or rats (37) with hepatic ChREBP deficiency. One possible cause of this discrepancy is suggested by a previous report in which knockdown of ChREBP was shown to reduce hepatic TG secretion in rats (37). We therefore determined the rate of hepatic TG secretion of control and *L-Chrebp*^−/−^ mice by measuring the rate of increase in plasma TG following administration of the lipoprotein lipase inhibitor, poloxamer 407. As shown in supplemental Fig. S3, the *L-Chrebp*^−/−^ mice indeed exhibited a 40% decrease in the rate of liver TG secretion relative to that of controls.

### Restoration of nSREBP-1c protein partially normalizes lipogenic genes in sucrose-refed *L-Chrebp*^−/−^ mice

**Fig. 5.**
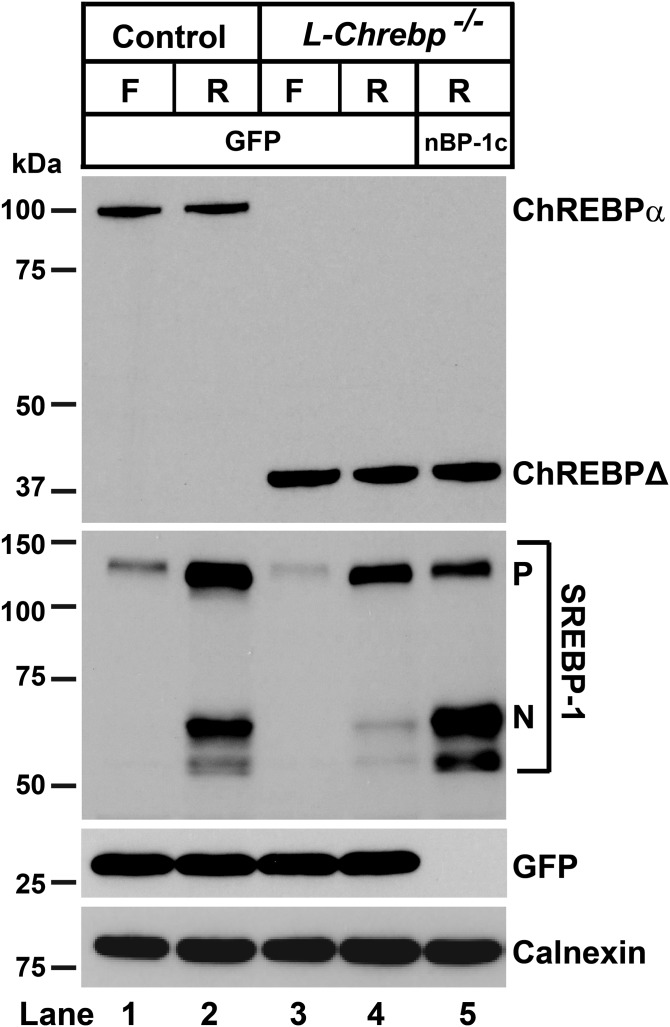
Immunoblot analysis of livers from sucrose-refed control and *L-Chrebp*^−/−^ mice treated with AAV-GFP or AAV-nSREBP-1c. Littermate control and *L-Chrebp*^−/−^ mice (same as those described in supplemental Table S3) were injected via the tail vein with recombinant AAV-CAG-GFP (GFP) or AAV-CAG-nSREBP-1c (nBP-1c) (2 × 10^11^ gene copies per mouse). Seven days after the injection, the mice were fasted for 12 h (F) or fasted for 12 h and then refed (R) with high-sucrose diet for 12 h prior to study. Liver whole-cell lysates were prepared individually, and equal amounts of protein from each mouse of the same group (four to six per group) were pooled. Aliquots (40 μg) of the pooled protein were subjected to SDS-PAGE and immunoblot analysis.

The reduction of hepatic SREBP-1c mRNA in sucrose-refed *L-Chrebp*^−/−^ mice together with the identification of ChoRE motifs in SREBP-1c promoter (51, 52) support the idea that SREBP-1c is a direct transcriptional target of ChREBP. This also raises the question of whether the blunted induction of lipogenic genes in *L-Chrebp*^−/−^ mice was caused by ChREBP deficiency itself or as a secondary effect of reduced SREBP-1c levels. To address this question, we tested to determine whether restoration of nSREBP-1c in the livers of *L-Chrebp*^−/−^ mice could normalize the expression of lipogenic genes in response to refeeding. For this purpose, we administered recombinant AAV encoding amino acids 1-456 of mouse SREBP-1c, which corresponds to the native nSREBP-1c produced by proteolytic processing, to *L-Chrebp*^−/−^ mice. Seven days after the injection of AAV-GFP or AAV-nSREBP-1c (nBP-1c), the mice were subjected to fasting and refeeding with high-sucrose diet. As shown in **Fig. 5**, nSREBP-1c protein in the livers of refed *L-Chrebp*^−/−^ mice was markedly reduced relative to that of refed control mice (Fig. 5, lane 2 vs. lane 4), reproducing the results of Fig. 3. AAV-nSREBP-1c injection restored nSREBP-1c protein in the livers of *L-Chrebp*^−/−^ mice to levels significantly higher than that of control mice (Fig. 5, lane 2 vs lane 5).

**Fig. 6.**
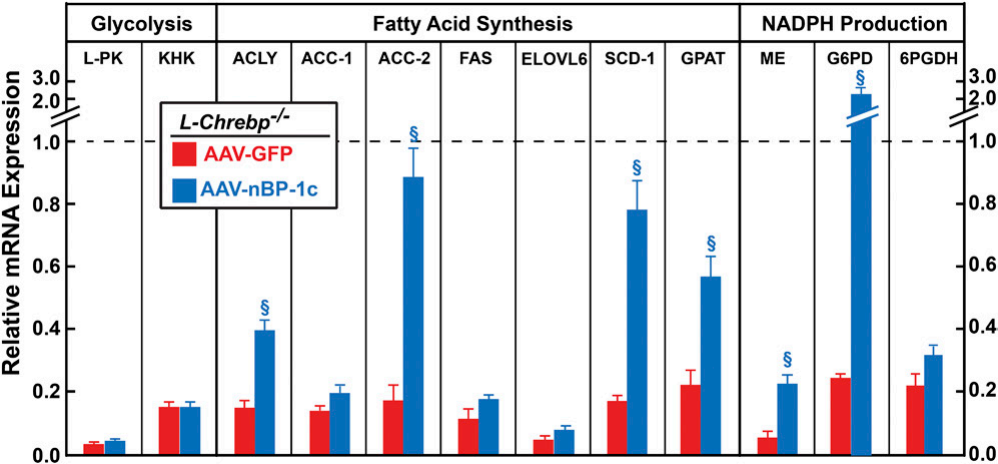
Relative gene expression in the livers from sucrose-refed control and *L-Chrebp*^−/−^ mice treated with AAV-GFP or AAV-nSREBP-1c. The mice used here were the same as those in Fig. 5. Total liver RNAs were prepared and subjected to real-time PCR analysis with ApoB as the invariant control. For brevity, only the sucrose-refed groups were shown here, and the mRNA expression was plotted as the amount relative to that in livers of sucrose-refed control mice, which was arbitrarily defined as 1.0 and indicated as a dotted line. Each bar represents the mean ± SEM of values from four to six mice per group. ^§^*P* < 0.01 denotes the level of statistical significance between sucrose-refed *L-Chrebp*^−/−^ mice injected with GFP or nBP-1c. Additional mRNA values from this experiment are shown in supplemental Table S3. G6PDH, glucose-6-phosphate dehydrogenase.

**Figure 6** shows the relative gene expression in the livers of AAV-GFP- or AAV-nSREBP-1c-injected sucrose-refed *L-Chrebp*^−/−^ mice. For clarity, mRNA values are normalized to refed control mice, which are set to 1.0. Restoration of nSREBP-1c in refed *L-Chrebp*^−/−^ mice did not normalize the mRNA levels of glycolytic genes (L-PK and KHK), which are direct targets of ChREBP, but not of SREBP-1c.

Relative to those of controls, mRNAs encoding enzymes for FA synthesis and NADPH production were all reduced by 75% to 95% in the livers of AAV-GFP-injected *L-Chrebp*^−/−^ mice (Fig. 6). In the livers of AAV-nSREBP-1c-injected *L-Chrebp*^−/−^ mice, only acetyl-CoA carboxylase (ACC)-2, SCD-1, and G6PD mRNAs were restored to levels comparable to those of sucrose-refed control mice. The reduced expression of other mRNAs encoding lipogenic genes were either partially restored [ATP citrate lyase (ACLY), glycerol-3-phosphate acyltransferase (GPAT), and malic enzyme (ME)] or were not restored by AAV-nSREBP-1c [ACC-1, FAS, long-chain fatty acyl elongase 6 (ELOVL6), and 6-phosphogluconate dehydrogenase (6PGDH)]. These data indicate that, in the absence of ChREBP, SREBP-1c alone is not sufficient to mediate the full refeeding-induced lipogenic response.

### ChREBP fails to restore lipogenic gene expression in sucrose-refed *L-Scap*^−/−^ mice

**Fig. 7.**
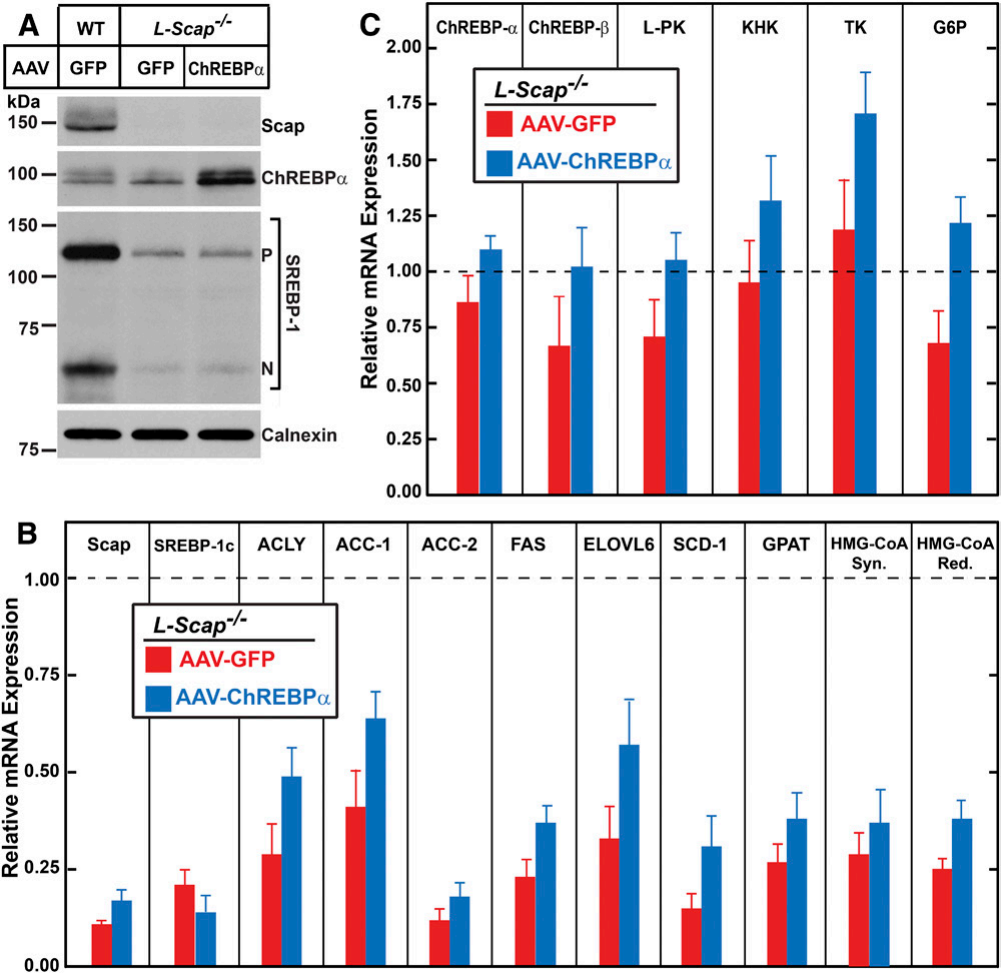
Overexpression of ChREBP-α fails to normalize the expression of lipogenic genes in the livers of sucrose-refed *L-Scap*^−/−^ mice. Littermate control and *L-Scap*^−/−^ mice (male, 6 months of age) were injected via the tail vein with recombinant AAV-TBG-GFP (GFP) or AAV-TBG-ChREBP-α (1 × 10^12^ gene copies per mouse). Two weeks after the injection, the mice were fasted for 12 h and then refed with high-sucrose diet for 12 h prior to study. A: Equal amounts of liver protein from each mouse of the same group (five to six per group) were pooled and subjected to immunoblot analysis. B, C: Total liver RNAs were prepared and subjected to real-time PCR analysis with ApoB as the invariant control. The mRNA expression was plotted as the amount relative to that in the livers of control mice, which was arbitrarily defined as 1.0 and indicated as a dotted line. Each bar represents the mean ± SEM of values from five to six mice per group.

We have shown previously that the postprandial lipogenic response is completely abolished when the hepatic activity of all three SREBPs is absent as a result of Scap deficiency (*L-Scap*^−/−^ mice) (20–22). In light of the role of ChREBP in this process demonstrated above, we next determined whether ChREBP and ChREBP-regulated genes are altered in the livers of sucrose-refed *L-Scap*^−/−^ mice. Relative to those of control mice, SREBP-1c and lipogenic mRNAs were all reduced in the livers of *L-Scap*^−/−^ mice, as shown previously (20–22) (**Figs. 7A, B**). Hepatic levels of ChREBP-α, ChREBP-β, and ChREBP-regulated glycolytic (L-PK, KHK, TK, and G6P) mRNAs, on the other hand, were not affected by Scap deficiency (Fig. 7C). Furthermore, even when AAV-ChREBP-α administration to *L-Scap*^−/−^ mice increased ChREBP-α protein to levels significantly higher than those of control mice (Fig. 7A, lane 3), the refeeding-induced lipogenic response was not restored (Fig. 7B). Therefore, in the absence of SREBP activity, ChREBP alone is not sufficient to mediate the sucrose-induced lipogenic response.

To verify the efficacy of AAV-ChREBP-α, we administered the AAV-ChREBP-α to *L-Chrebp*^−/−^ mice. As shown in supplemental Fig. S4A, AAV-ChREBP-α injection restored ChREBP-α protein in the livers of sucrose-refed *L-Chrebp*^−/−^ mice to levels of about 50% of those in sucrose-refed control mice. This partial restoration of ChREBP-α protein was sufficient to normalize the mRNA levels of ChREBP-α-, ChREBP-β-, and ChREBP-regulated genes encoding key enzymes of glucose metabolism (supplemental Fig. S4B). The expression of SREBP-1c mRNA and protein as well as lipogenic mRNAs were also restored by AAV-ChREBP-α (supplemental Figs. S4A, C). These data further support the hypothesis that the postprandial induction of SREBP-1c requires ChREBP, and that both transcription factors are required to coordinate the postprandial lipogenic response.

## DISCUSSION

The current results demonstrate an essential role for ChREBP in regulating glycolysis and lipogenesis in mouse liver and the interdependence of ChREBP and SREBP-1c for the coordinated maximal induction of lipogenesis by carbohydrates. Hepatic deletion of ChREBP reduced the basal levels of glycolytic and lipogenic mRNAs and prevented the induction of these genes in response to sucrose. Virally mediated restoration of nSREBP-1c in *L-Chrebp*^−/−^ mice normalized the sucrose-induced stimulation of a subset of lipogenic genes, while not affecting any glycolytic genes. Conversely, in *L-Scap*^−/−^ mice lacking all SREBPs, ChREBP failed to normalize the postprandial induction of lipogenic genes. Together with previous studies, these results demonstrate that SREBP-1c and ChREBP are both required for the coordinated induction of glycolytic and lipogenic mRNAs in response to excess carbohydrates. Whereas SREBP-1c mediates insulin’s induction of lipogenic genes, ChREBP mediates glucose’s induction of both glycolytic and lipogenic genes. This provides a potential mechanism to ensure that the liver will not synthesize FAs unless insulin and glucose are both present.

Mice with germline ChREBP deficiency became moribund when fed a high-sucrose diet, although they consumed the same amount of starch-containing chow diet as control littermates (Fig. 2). The liver-specific *L-Chrebp*^−/−^ mice, in marked contrast, can adapt and tolerate sucrose feeding, which is consistent with a previous report (37) that showed that ASO-mediated knockdown of ChREBP in liver and adipose tissue led to only a modest reduction of sucrose intake in rats. Likewise, a recent study showed that adipose-specific deletion of ChREBP did not result in sucrose-intolerance (53). These data suggest that ChREBP deficiency in liver or adipose tissue does not lead to systemic sucrose/fructose intolerance. Given that ChREBP is broadly expressed in metabolically active tissues, its activities in other tissues, such as brain, intestine, or pancreatic islets, are likely important for preventing systemic fructose toxicity (28–31). Indeed, a recent study has revealed that fructose tolerance requires intestinal, but not hepatic, ChREBP (46).

The current data support the notion that SREBP-1c and ChREBP have overlapping yet distinct roles in regulating glucose and lipid metabolism. ChREBP, but not SREBP-1c, is required for the basal and carbohydrate-induced expression of glycolytic genes, such as *L-Pk*, *Khk*, and *Tk*. The full induction of the hepatic lipogenic program by insulin and glucose, however, requires both SREBP-1c and ChREBP. The presence of both SRE and ChoRE motifs in the promoters of most lipogenic genes (51, 52, 54) is consistent with the requirement of both factors for the full transcriptional induction. The molecular mechanism by which SREBP-1c and ChREBP dually activate transcription from the same promoter regions remains unknown. Possibly, cofactors or cofactor complexes interact with both SREBP-1c and ChREBP when bound to their regulatory elements in an insulin- and/or glucose-dependent manner. For example, PPAR-γ coactivator 1β (PGC-1β) has been reported to be involved in the coactivation of both SREBP-1c and ChREBP (55, 56).

These data reveal hitherto unappreciated regulation of SREBP-1c by ChREBP. When mice were fasted and sucrose-refed, both SREBP-1c mRNA and nSREBP-1 protein were reduced in the livers of *L-Chrebp*^−/−^ mice relative to those of controls (Figs. 3–6). The reduced expression of SREBP-1c mRNA and protein was normalized when ChREBP-α was restored in *L-Chrebp*^−/−^ mice by AAV-ChREBP-α (supplemental Fig. S3). This restoration was likely mediated by direct binding of ChREBP to the ChoRE motifs in the promoter region of *Srebp-1c* to increase SREBP-1c mRNA and precursor protein (51, 52). The amount of nSREBP-1 protein was reduced out of proportion to the decrease of SREBP-1c mRNA in *L-Chrebp*^−/−^ livers, suggesting that ChREBP deficiency might also affect nSREBP-1c protein, independent of its effect on SREBP-1c mRNA. The reduction of SREBP-1 in *L-Chrebp*^−/−^ livers, however, was not associated with significant changes in SREBP-2 or key regulators of SREBP proteolysis (i.e., Insigs and Scap) (Fig. 3), suggesting that ChREBP deficiency did not cause a general blockade of SREBP processing or transcriptional activation. Future studies are needed to elucidate whether ChREBP regulates SREBP-1c posttranscriptionally, either by affecting the proteolytic processing of SREBP-1c precursor or by other mechanism(s).

A recent study has shown that SREBP-2 and SREBP-2-regulated cholesterol biosynthetic mRNAs were elevated in the livers of germline *Chrebp*^−/−^ mice fed with a high-fructose diet (57). However, consistent with our previous (29) and current data (Fig. 2), Zhang et al. (57) also noted that the germline *Chrebp*^−/−^ mice were intolerant of high-fructose diet and lost ∼20% of their body weight following 2 weeks of fructose exposure. Thus, the changes in hepatic SREBP-2 in fructose-fed germline *Chrebp*^−/−^ mice may be a secondary effect of fructose-related toxicity rather than a primary effect of ChREBP deficiency on SREBP-2.

The regulation of SREBP-1c by ChREBP adds another layer of control to the complex network that coordinates the action of insulin and glucose on glycolysis and lipogenesis. In addition to SREBP-1c and ChREBP, liver X receptors (LXRs) are also required for normal rates of lipogenesis. LXRs are members of the nuclear receptor family of transcription factors that are activated by oxysterols and cholesterol intermediates (58, 59). LXR is required for basal expression and for insulin’s activation of SREBP-1c transcription (17, 60). LXR also directly activates the transcription of ChREBP (61) and some lipogenic genes (58, 59).

Previous studies (40, 62, 63) support the hypothesis that the cholesterol biosynthetic pathway, which is controlled by Scap/SREBP-2, produces endogenous sterol ligands necessary for the activation of LXR. Accordingly, because ChREBP is a transcriptional target of LXR, ChREBP mRNA levels were reduced in SREBP-2-deficient livers (40). However, this fall in ChREBP expression could be attributed to either a reduction in the synthesis of sterol ligands, reducing LXR activity, or to reductions in nSREBPs themselves. Similarly, under ad libitum chow-fed conditions, hepatic mRNA levels of LXR-regulated genes, such as *Srebp-1c*, *Chrebp-α*, *Chrebp-β*, *Fas*, *Abc-g5* (*P* = 0.06), and *Abc-g8*, were all reduced in *L-Scap*^−/−^ mice (supplemental Fig. S4). Administration of a synthetic LXR agonist, T0901317, to *L-Scap*^−/−^ mice restored hepatic ABC-G5 and ABC-G8 mRNAs to levels similar to those in control mice, and partially restored SREBP-1c mRNA. The same T0901317 treatment, however, failed to restore the hepatic expression of *Chrebp-α* or *Chrebp-β* (supplemental Fig. S4). This suggests that SREBP activity, independent of producing LXR agonists, supports ChREBP expression, at least under certain dietary conditions. Indeed, T0901317-mediated induction of ChREBP mRNA was also blunted in the livers of *Srebp-1c*^−/−^ mice (data not shown). Thus, LXR’s regulation of ChREBP is mediated by the direct action of LXR on the *Chrebp* promoter (61) as well as the indirect LXR-mediated activation of SREBP-1c. This is similar to the LXR-regulation of ACC and FAS (20, 58, 59).

LXR or SREBP activity, however, does not appear to be required for the sucrose-mediated activation of ChREBP and its glycolytic target genes. As shown in Fig. 7, the hepatic expression of *Chrebp* and its glycolytic target genes was not different between sucrose-refed control and *L-Scap*^−/−^ mice. The sucrose-mediated induction of *Chrebp* and its glycolytic target genes was also comparable between the livers of control and LXR-deficient mice (64). In addition, in the absence of functional ChREBP, overexpression of nSREBP-1c failed to stimulate the transcription of ChREBP (supplemental Table S3). Thus, although LXR and SREBP can activate ChREBP expression, neither appears to be required for the sucrose-mediated activation of ChREBP and its glycolytic target genes. In contrast, the induction of *Srebp-1c* and its lipogenic target genes by sucrose-refeeding was abolished in the absence of Scap [Fig. 7 and (20, 21)] or LXR (60, 64). These data are consistent with the combined requirement of LXR and SREBPs themselves for the insulin-mediated activation of SREBP-1c transcription (17). Under the sucrose-refed condition, ChREBP likely regulates itself in a feed-forward fashion (7, 9, 32, 49). The full induction of the lipogenic mRNAs, on the other hand, requires the coordinated actions of ChREBP, LXR, and SREBP-1c.

Recently, Jois et al. (65) reported that hepatic-specific deletion of a 0.5 kb region containing exon 1a of ChREBP-α impairs glucose homeostasis and hepatic insulin sensitivity. Intriguingly, in this liver-ChREBP knockout model, the expression of canonical transcription targets of ChREBP, such as *L-Pk*, *Acc-1*, and *Fas*, was not reduced (65). This is in sharp contrast with the dramatic decrease of these glycolytic and lipogenic mRNAs observed in the livers of our germline *Chrebp*^−/−^ (29) or *L-Chrebp*^−/−^ mice [data presented here and in (46)]. One possible explanation for this discrepancy might be the difference in the targeting approach. In our *L-Chrebp*^−/−^ model, Cre-mediated recombination removed exons 9-15, which encode various functional domains of ChREBP, such as the proline-rich domain and the DNA-binding domain present in both ChREBP-α and -β isoforms. In contrast, Jois et al. (65) only deleted a 0.5 kb region containing exon 1a of ChREBP-α, leaving the upstream promoter region and exon 1b of the ChREBP-β intact. Thus, it is possible that, in the liver-ChREBP knockout model reported by Jois et al. (65), the constitutively active ChREBP-β isoform is still produced and is able to partially compensate for the loss of ChREBP-α (32, 46). The impairment of glucose and insulin sensitivity noted by Jois et al. (65) suggests that ChREBP-α and -β may have nonoverlapping functions and further research in this area is warranted.

**Fig. 8.**
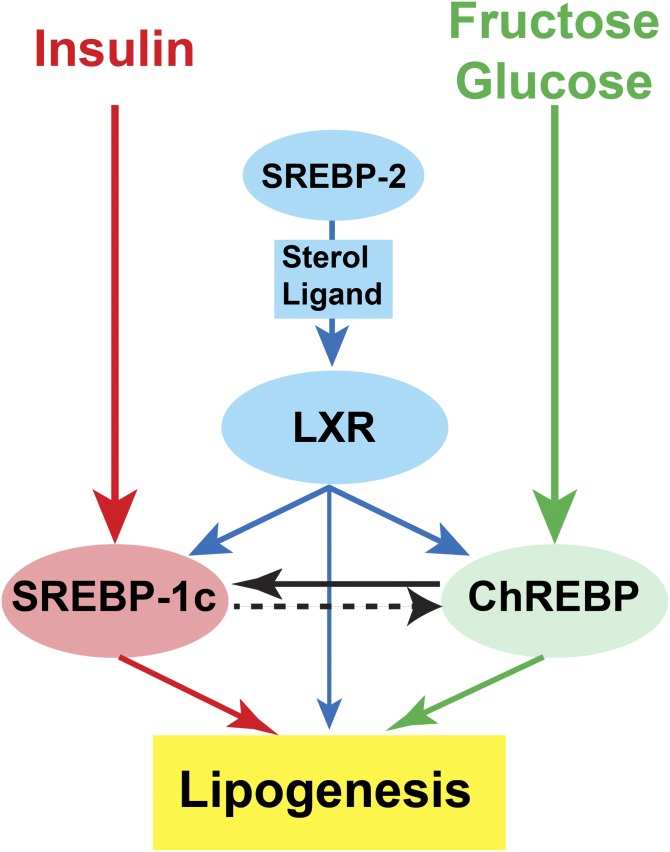
Regulation of hepatic lipogenesis requires the concerted actions of ChREBP, LXR, and SREBP-1c. This schematic depicts the synergistic action of insulin and fructose/glucose in promoting hepatic lipogenesis. Insulin activates the transcription of SREBP-1c mRNA as well as the proteolytic processing of SREBP-1c protein, whereas fructose/glucose activates ChREBP by multiple mechanisms. The insulin-mediated activation of SREBP-1c transcription acts through LXR and SREBPs themselves. Although LXR regulates lipogenesis primarily by increasing SREBP-1c mRNA, it can also directly activate the promoters of *Chrebp* and some lipogenic genes. SREBP-2, which regulates genes of cholesterol biosynthesis, controls the production of endogenous sterol ligands necessary for LXR to activate the transcription of SREBP-1c, ChREBP, and lipogenic genes. ChREBP also directly regulates SREBP-1c expression and, at least under certain conditions, SREBP-1c can also regulate ChREBP. Together, SREBP-1c, LXR, and ChREBP are required for maximal induction of postprandial hepatic lipogenesis.

In summary, together with previous reports (3–7, 9), the current study suggests that the maximal postprandial induction of lipogenesis requires the synergistic action of both insulin-dependent activation of LXR/SREBP-1c and fructose/glucose-dependent activation of ChREBP (**Fig. 8**). The relative importance of ChREBP is highest in the setting of excess fructose ingestion (7, 9, 35, 49). Inhibition of SREBP activation, which simultaneously blocks all three key regulators of hepatic lipogenesis, SREBP, LXR, and ChREBP, has therapeutic potential for treatment of fatty liver disease (22).

## Supplementary Material

Supplemental Data
