# Supplementary material for: Interplay between ChREBP and SREBP-1c coordinates postprandial glycolysis and lipogenesis in livers of mice
Source: J Lipid Res. 2018 Jan 15;59(3):475–87. doi: 10.1194/jlr.M081836 (PMC5832931; doi:10.1194/jlr.M081836)
Supplement: Supplemental Data [file supp_59_3_475__index.html]

Interplay between ChREBP and SREBP-1c Coordinates Postprandial Glycolysis and Lipogenesis in Livers of Mice — Interplay between ChREBP and SREBP-1c coordinates postprandial glycolysis and lipogenesis in livers of mice — Supplemental Data 

# Interplay between ChREBP and SREBP-1c coordinates postprandial glycolysis and lipogenesis in livers of mice

## Supplemental Data

- Supplemental data (.pdf, 518 KB) - Supplemental data
