# Supplementary material for: Interplay between ChREBP and SREBP-1c coordinates postprandial glycolysis and lipogenesis in livers of mice
Source: J Lipid Res. 2018 Jan 15;59(3):475–87. doi: 10.1194/jlr.M081836 (PMC5832931; doi:10.1194/jlr.M081836)
Supplement: Supplemental Data [file 10.1194_M081836_jlr.M081836-1.pdf]

SUPPLEMENTAL DATA

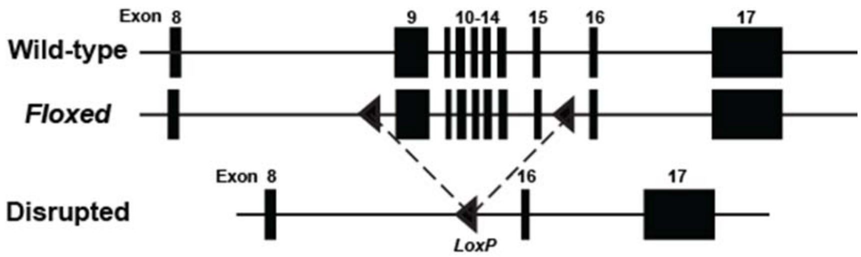

Supplemental Fig. S1. Strategy for conditional gene-targeting of *Chrebp*. Mice carrying the conditional *floxed Chrebp* allele (*Chrebp*$^{f/f}$) were generated by inserting one *loxP* site upstream of exon 9 and another *loxP* site immediately downstream of exon 15. Cre-mediated recombination between the *loxP* sites deletes the region spanning exons 9 to 15, which encodes various functional domains of ChREBP such as the proline-rich domain and the basic helix-loop-helix leucine zipper DNA-binding domain present in both ChREBP-α and ChREBP-β isoforms.

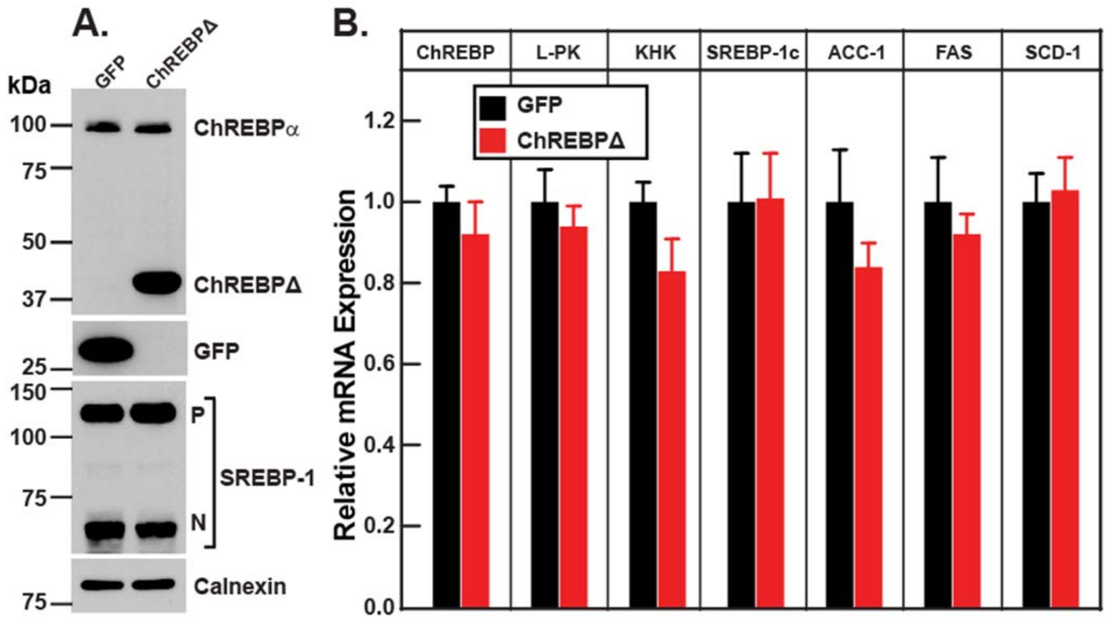

Supplemental Fig. S2. Overexpression ChREBPΔ in wild-type mice does not affect the expression of ChREBP or ChREBP-regulated genes. Wild-type C57BL6/J mice (male, 10 weeks of age) were injected via the tail vein with recombinant AAV-CAG-GFP or AAV-CAG-ChREBPΔ ($1x10^{11}$ gene copies per mouse). Ten days after the injection, the mice were fasted 12 h and then refed high-sucrose diet for 12 h prior to study. (**A**) Liver whole cell lysates were prepared individually, and equal amounts of protein from each mouse of the same group (6 per group) were pooled. Aliquots (40 µg) of the pooled protein were subjected to SDS-PAGE and immunoblot analysis. (**B**) Total liver RNAs were prepared and subjected to real-time PCR analysis with ApoB as the invariant control. Each bar represents mean ± SEM of values from 6 mice per group. The mRNA level was calculated as the amount relative to that in livers of GFP-injected mice, which was arbitrarily defined as 1.0.

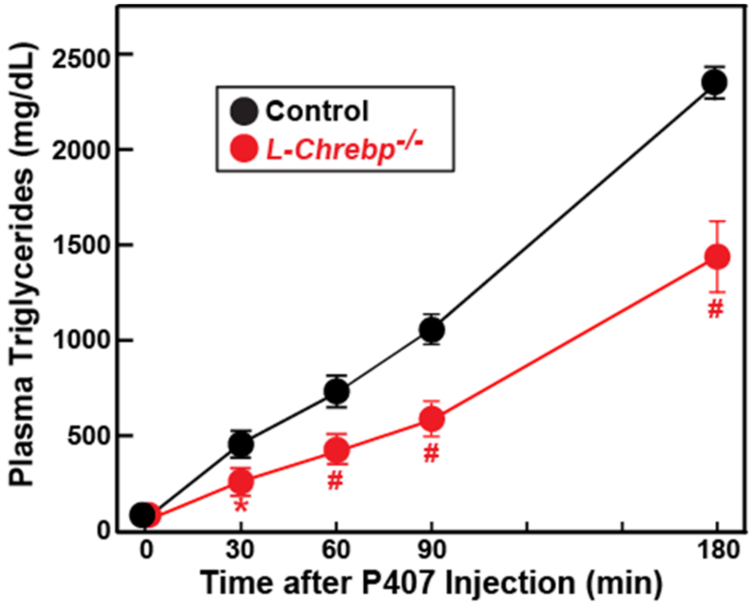

Supplemental Fig. S3. Liver ChREBP deficiency reduces hepatic TG secretion. Littermate control and *L-Chrebp*$^{-/-}$ mice (female, six months of age) were fasted for 3 h and then IP injected with 1 mg/g body weight of 7.5% (in saline) Poloxamer 407 (Cat. No. 16758, Sigma). Blood samples were obtained immediately prior to injection (time 0), and at 30, 60, 90, and 180 min following injection. Plasma TG concentrations were measured and plotted. Each value represents the mean ± SEM of data from 5 animals. *p<0.05 and $^{\#}$p<0.01 denote the level of statistical significance (two-tailed Student's t test) between control and *L-Chrebp*$^{-/-}$ mice.

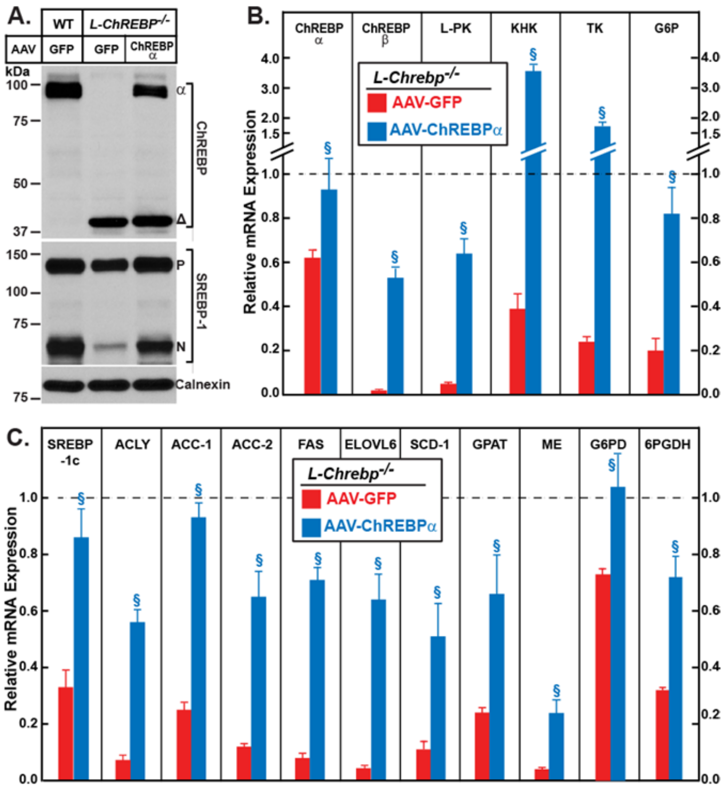

Supplemental Fig. S4. Restoration of ChREBP-α normalizes the expression of glycolytic and lipogenic genes in sucrose-refed *L-Chrebp*$^{-/-}$ mice. Littermate control and *L-Chrebp*$^{-/-}$ mice (male, 6-7 months of age) were injected via the tail vein with recombinant AAV-TBG-GFP (GFP) or AAV-TBG-ChREBP-α ($1x10^{12}$ gene copies per mouse). Two weeks after the injection, the mice were fasted for 12 h and then refed with high-sucrose diet for 12 h prior to study. (**A**). Equal amounts of liver protein from each mouse of the same group (4 per group) were pooled and subjected to immunoblot analysis. (**B-C**).Total liver RNA was prepared and subjected to real-time PCR analysis with ApoB as the invariant control. The mRNA expression was plotted as the amount relative to that in livers of control mice, which was arbitrarily defined as 1.0 and indicated as a dotted line. Each bar represents mean ± SEM of values from 4 mice per group. $^{\S}p<0.01$ denote the level of statistical significance between refed *L-Chrebp*$^{-/-}$ mice injected with GFP or ChREBP-α.

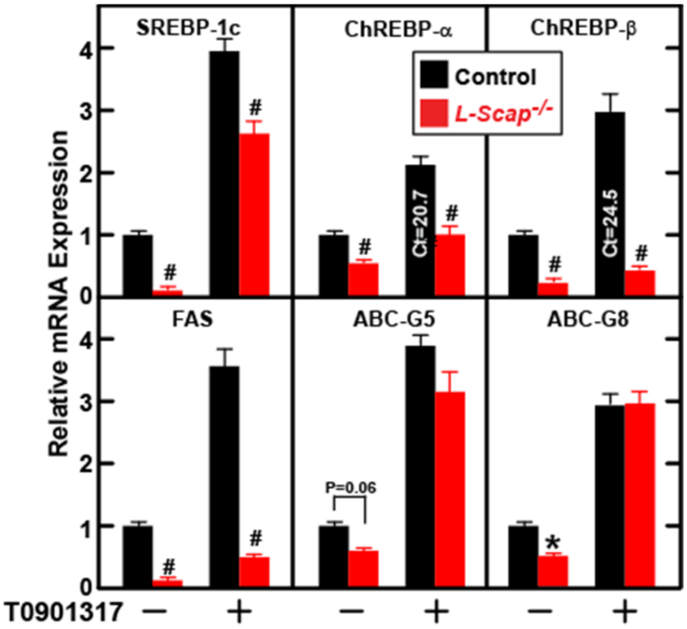

Supplemental Fig. S5. Relative gene expression in livers of control and *L-Scap*$^{-/-}$ mice fed chow diet supplemented with LXR agonist T0901317. Control and *L-Scap*$^{-/-}$ littermates (female, 3-4 months of age) were fed an *ad libitum* chow diet without (-) or with (+) 0.025% of LXR agonist T0901317 for 2 days prior to study. Total liver RNAs were prepared and subjected to real-time PCR analysis with ApoB as the invariant control. Each bar represents mean ± SEM of values from 5 mice per group. The mRNA level was calculated as the amount relative to that in livers of chow-fed control mice, which was arbitrarily defined as 1.0. *$p<0.05$ and $^{\#}p<0.01$ denote the level of statistical significance (two-tailed Student's t test) between control and *L-Scap*$^{-/-}$ mice under the same dietary condition.

Supplemental Table S1. Sequences of real-time PCR primers

mRNA	Forward (top) and reverse (bottom) primers
36B4	5’-CACTGGTCTAGGACCCGAGAAG-3’<br>5’-GGTGCCTCTGGAGATTTTCG-3’
ApoB	5’-CGTGGGCTCCAGCATTCTA-3’<br>5’-TCACCAGTCATTTCTGCCTTTG-3’
ChREBP-total	5’-GCCTCCGCCAGACCTCACTG-3’<br>5’-AGTGCTGAGTTGGCGAAGGG-3’
ChREBP-α	5’-CGACACTCACCCACCTCTTC-3’<br>5’-TTGTTCAGCCGGATCTTGTC-3’
ChREBP-β	5’-TCTGCAGATCGCGTGGAG-3’<br>5’-CTTGTCCCGGCATAGCAAC-3’
L-Pyruvate kinase	5’-GGCATCGAAAGTGGAAAGCT-3’<br>5’-GCCAGCCTGTCACCACAAT-3’
Glucokinase	5’-CCGTGATCCGGGAAGAGAA-3’<br>5’-GGGAAACCTGACAGGGATGAG-3’
Ketohexokinase	5’-CGTGAGGAGCTCTTCCAGTTG-3’<br>5’-CAGGTGCTTGGCCACATCT-3’
Triose kinase	5’-GCCGGGTAGCACTTCTGTCA-3’<br>5’-TTAGCATCCCTTTCCCGATAAA-3’
Glucose-6-phosphatase	5’-TGGGCAAAATGGCAAGGA-3’<br>5’-TCTGCCCCAGGAATCAAAAAT-3’
Scap	5’-GGCTGATCCATGGTCACTTT-3’<br>5’-AGCAGGCTAAGATGCAGAGTC-3’
SREBP-1a	5’-GGCCGAGATGTGCGAACT-3’<br>5’-TTGTTGATGAGCTGGAGCATGT-3’
SREBP-1c	5’-GGAGCCATGGATTGCACATT-3’<br>5’-GGCCCGGGAAGTCACTGT-3’
SREBP-2	5’-GCGTTCTGGAGACCATGGA-3’<br>5’-ACAAAGTTGCTCTGAAAACAAATCA-3’
Insig-1	5’-TCACAGTGACTGAGCTTCAGCA-3’<br>5’-TCATCTTCATCACACCCAGGAC-3’
Insig-2	5’-CGGAAGATGCTGGAACCTGA-3’<br>5’-TGTGCTCTCCATACGCTCTCC-3’
ATP citrate lyase	5’-GCCAGCGGGAGCACATC-3’<br>5’-CTTTGCAGGTGCCACTTCATC-3’
Acetyl-CoA synthetase 2	5’-GCTGCCGACGGGATCAG-3’<br>5’-TCCAGACACATTGAGCATGTCAT-3’
Acetyl-CoA carboxylase 1	5’-TGGACAGACTGATCGCAGAGAAAG-3’<br>5’-TGGAGAGCCCCACACACA-3’
Acetyl-CoA carboxylase 2	5’-GGGCTCCCTGGATGACAAC-3’<br>5’-GCTCTTCCGGGAGGAGTTCT-3’
Fatty acid synthase	5’-GCTGCGGAAACTTCAGGAAAT-3’<br>5’-AGAGACGTGTCACTCCTGGACTT-3’
Long chain fatty acyl elongase 6	5’-TGTACGCTGCCTTTATCTTTGG-3’<br>5’-GCGGCTTCCGAAGTTCAA-3’
Stearoyl-CoA desaturase-1	5’-CCGGAGACCCCTTAGATCGA-3’<br>5’-TAGCCTGTAAAAGATTTCTGCAAACC-3’
Glycerol-3-phosphate acyltransferase	5’-CAACACCATCCCCGACATC-3’<br>5’-GTGACCTTCGATTATGCGATCA-3’

Malic enzyme	5'-GCCGGCTCTATCCTCCTTTG-3'<br>5'-TTTGTATGCATCTTGCACAATCTTT-3'
Glucose-6-phosphate dehydrogenase	5'-GAACGCAAAGCTGAAGTGAGACT-3'<br>5'-TCATTACGCTTGCACTGTTGGT-3'
6-phosphogluconate dehydrogenase	5'-AGGCCCTCTATGCTTCCAAGA-3'<br>5'-CTCAGTGGCTGCCTGTCTGA-3'
HMG-CoA synthase	5'-GCCGTGAACTGGGTCGAA-3'<br>5'-GCATATATAGCAATGTCTCCTGCAA-3'
HMG-CoA reductase	5'-CTTGTGGAATGCCTTGTGATTG-3'<br>5'-AGCCGAAGCAGCACATGAT-3'
PPAR-α	5'-ACAAGGCCTCAGGGTACCA-3'<br>5'-GCCGAAAGAAGCCCTTACAG-3'
PPAR-γ	5'-CACAATGCCATCAGGTTTGG-3'<br>5'-GCTGGTCGATATCACTGGAGATC-3'
ABC-G5	5'-TGGATCCAACACCTCTATGCTAAA-3'<br>5'-GGCAGGTTTTCTCGATGAACTG-3'
ABC-G8	5'-TGCCCACCTTCCACATGTC-3'<br>5'-ATGAAGCCGGCAGTAAGGTAGA-3'

Note: ChREBP-total primer detects the wild-type ChREBP, but not the aberrant ChREBP. ChREBP-α and-β primers detect both wild-type and aberrant ChREBP-α and -β isoforms.

Supplemental Table S2A. Phenotypic parameters and liver mRNA levels of control and *L-Chrebp*$^{-/-}$ mice fed chow diet *ad libitum*

	Control	*L-Chrebp*$^{-/-}$
	**Nonfasted**	**Nonfasted**
**Phenotypic Parameters**		
Number of mice	4	4
Body weight (g)	34.8 ± 1.0	38.4 ± 0.2*
Liver glycogen content (mg/g)	46 ± 9	79 ± 9*
Liver cholesterol content (mg/g)	1.9 ± 0.1	1.7 ± 0.1*
Liver triglyceride content (mg/g)	19 ± 3	52 ± 3#
Plasma cholesterol (mg/dL)	156 ± 11	118 ± 1*
Plasma triglyceride (mg/dL)	190 ± 9	232 ± 23
Plasma aspartate transaminase (U/L)	41 ± 5	52 ± 1
Plasma alanine transaminase (U/L)	41 ± 2	65 ± 2#
Plasma insulin (ng/mL)	1.1 ± 0.0	1.3 ± 0.3
Plasma glucose (mg/dL)	264 ± 10	227 ± 5*
**Liver mRNAs**		
ApoB raw Ct number	17.6 ± 0.0	17.6 ± 0.1
**ChREBP and glucose metabolism**		
ChREBP-total	1.0 ± 0.1	0.0 ± 0.0#
ChREBP-α	1.0 ± 0.1	0.4 ± 0.0#
ChREBP-β	1.0 ± 0.2	0.0 ± 0.0#
L-Pyruvate Kinase	1.0 ± 0.1	0.1 ± 0.0#
Glucokinase	1.0 ± 0.1	1.5 ± 0.2*
Ketohexokinase	1.0 ± 0.1	0.5 ± 0.0#
Triose kinase	1.0 ± 0.1	0.4 ± 0.0#
Glucose-6-phosphatase	1.0 ± 0.1	0.7 ± 0.0*
**SREBP pathway**		
Scap	1.0 ± 0.1	0.8 ± 0.0*
SREBP-1a	1.0 ± 0.0	1.0 ± 0.1
SREBP-1c	1.0 ± 0.1	0.9 ± 0.1
SREBP-2	1.0 ± 0.0	1.3 ± 0.1
Insig-1	1.0 ± 0.1	0.7 ± 0.1
Insig-2	1.0 ± 0.1	0.8 ± 0.0
**Fatty acid synthesis**		
ATP citrate lyase	1.0 ± 0.1	0.5 ± 0.0#
Acetyl-CoA synthetase 2	1.0 ± 0.1	0.6 ± 0.1#
Acetyl-CoA carboxylase 1	1.0 ± 0.1	0.7 ± 0.0*
Acetyl-CoA carboxylase 2	1.0 ± 0.2	0.2 ± 0.0#
Fatty acid synthase	1.0 ± 0.1	0.3 ± 0.0#
Long chain fatty acyl elongase 6	1.0 ± 0.1	0.3 ± 0.0#
Stearoyl-CoA desaturase-1	1.0 ± 0.2	0.4 ± 0.0#
Glycerol-3-phosphate acyltransferase	1.0 ± 0.1	0.6 ± 0.0#
Malic enzyme	1.0 ± 0.1	0.1 ± 0.0#
Glucose-6-phosphate dehydrogenase	1.0 ± 0.1	0.8 ± 0.0
6-phosphogluconate dehydrogenase	1.0 ± 0.1	0.7 ± 0.0*
**Cholesterol synthesis**		
HMG-CoA synthase	1.0 ± 0.1	0.9 ± 0.1
HMG-CoA reductase	1.0 ± 0.1	0.8 ± 0.1

**Nuclear receptors**		
PPAR-α	1.0 ± 0.1	0.7 ± 0.0
PPAR-γ	1.0 ± 0.1	1.0 ± 0.1

Supplemental Table S2B. Phenotypic parameters and liver mRNA levels of control and *L-Chrebp*$^{-/-}$ mice subjected to fasting and refeeding with high-sucrose diet

	Control		*L-Chrebp*$^{-/-}$	
	**Fasted**	**Refed**	**Fasted**	**Refed**
**Phenotypic Parameters**				
Number of mice	4	4	4	4
Body weight (g)	30.6 ± 1.3	37.5 ± 1.8	30.6 ± 1.0	35.2 ± 1.5
Liver glycogen content (mg/g)	1.8 ± 0.8	101 ± 7	2.3 ± 1.0	122 ± 5*
Liver cholesterol content (mg/g)	2.3 ± 0.3	1.9 ± 0.1	2.8 ± 0.2	1.5 ± 0.1
Liver triglyceride content (mg/g)	95 ± 8	35 ± 5	167 ± 15#	43 ± 5
Plasma cholesterol (mg/dL)	129 ± 10	128 ± 4	87 ± 2#	81 ± 2#
Plasma triglyceride (mg/dL)	178 ± 9	164 ± 16	170 ± 13	172 ± 13
Plasma aspartate transaminase (U/L)	55 ± 13	40 ± 3	44 ± 11	43 ± 3
Plasma alanine transaminase (U/L)	48 ± 2	36 ± 3	70 ± 6*	60 ± 5#
Plasma insulin (ng/mL)	0.3 ± 0.1	2.8 ± 0.6	0.2 ± 0.1	1.9 ± 0.7
Plasma glucose (mg/dL)	156 ± 25	231 ± 13	125 ± 13	190 ± 12
**Liver mRNAs**				
ApoB raw Ct number	17.3 ± 0.0	17.6 ± 0.1	17.5 ± 0.1	17.6 ± 0.1
**ChREBP and glucose metabolism**				
ChREBP-total	1.0 ± 0.1	4.6 ± 0.2	0.0 ± 0.0#	0.0 ± 0.0#
ChREBP-α	1.0 ± 0.1	2.3 ± 0.2	0.4 ± 0.0#	1.0 ± 0.0#
ChREBP-β	1.0 ± 0.2	11 ± 0.2	0.0 ± 0.0#	0.0 ± 0.0#
L-Pyruvate kinase	1.0 ± 0.1	15 ± 1	0.2 ± 0.0#	0.4 ± 0.0#
Glucokinase	1.0 ± 0.3	16 ± 2	2.4 ± 1.1	28 ± 1#
Ketohexokinase	1.0 ± 0.2	4.5 ± 1.2	0.7 ± 0.1	1.1 ± 0.0*
Triose kinase	1.0 ± 0.3	7.1 ± 0.2	0.6 ± 0.3	1.0 ± 0.0#
Glucose-6-phosphatase	1.0 ± 0.1	7.9 ± 1.2	1.1 ± 0.1	1.6 ± 0.0#
**SREBP pathway**				
Scap	1.0 ± 0.1	2.5 ± 0.2	1.0 ± 0.0	1.3 ± 0.1#
SREBP-1a	1.0 ± 0.0	4.0 ± 0.1	1.1 ± 0.1	3.3 ± 0.2*
SREBP-1c	1.0 ± 0.1	46 ± 8	0.8 ± 0.0	26 ± 1*
SREBP-2	1.0 ± 0.0	2.5 ± 0.1	1.8 ± 0.1#	3.5 ± 0.3*
Insig-1	1.0 ± 0.2	10 ± 1	1.6 ± 0.4	11 ± 0
Insig-2	1.0 ± 0.1	0.8 ± 0.0	1.0 ± 0.1	0.6 ± 0.1
**Fatty acid synthesis**				
ATP citrate lyase	1.0 ± 0.1	52 ± 3	0.8 ± 0.1	5.7 ± 1.1#
Acetyl-CoA synthetase 2	1.0 ± 0.1	17 ± 2	1.0 ± 0.0	7.0 ± 1.6#
Acetyl-CoA carboxylase 1	1.0 ± 0.1	21 ± 1.7	0.8 ± 0.1	2.7 ± 0.1#
Acetyl-CoA carboxylase 2	1.0 ± 0.0	6.1 ± 0.7	0.9 ± 0.1	0.5 ± 0.2#
Fatty acid synthase	1.0 ± 0.1	67 ± 7	0.7 ± 0.0	5.7 ± 0.6#
Long chain fatty acyl elongase 6	1.0 ± 0.1	46 ± 8	0.3 ± 0.0#	1.6 ± 0.3#
Stearoyl-CoA desaturase-1	1.0 ± 0.4	4.5 ± 0.4	0.3 ± 0.1	0.5 ± 0.1#
Glycerol-3-phosphate acyltransferase	1.0 ± 0.1	9.0 ± 1.4	1.4 ± 0.1#	1.5 ± 0.2#
Malic enzyme	1.0 ± 0.1	9.2 ± 0.8	0.4 ± 0.1#	0.3 ± 0.1#
Glucose-6-phosphate dehydrogenase	1.0 ± 0.1	8.6 ± 1.2	1.4 ± 0.1*	1.8 ± 0.1#
6-phosphogluconate dehydrogenase	1.0 ± 0.1	13 ± 1	1.0 ± 0.0	2.3 ± 0.3#
**Cholesterol synthesis**				
HMG-CoA synthase	1.0 ± 0.1	8.8 ± 0.9	1.8 ± 0.2#	9.2 ± 1.5
HMG-CoA reductase	1.0 ± 0.0	11 ± 2	1.4 ± 0.1*	7.6 ± 1.3
**Nuclear receptors**				

PPAR-α	1.0 ± 0.1	0.5 ± 0.1	0.8 ± 0.0	0.4 ± 0.3
PPAR-γ	1.0 ± 0.1	1.4 ± 0.1	1.1 ± 0.1	1.4 ± 0.1

Control and *L-Chrebp*$^{-/-}$ littermates (male, 7 months of age) were subjected to fasting and refeeding. The nonfasted (N) group was maintained on *ad libitum* chow diet, the fasted (F) group was fasted 12 h, and the refed (R) group was fasted for 12 h and then refed a 60% (w/w) high-sucrose diet for 12 h prior to study. The starting times for the feeding regimens were staggered so that all mice were sacrificed at the same time, which was at the end of the dark cycle. For clarity, the results were presented in two separate Tables. Table S2A shows the data of nonfasted groups, whereas Table S2B shows the data of fasted and refed groups. Total RNA was isolated from livers and subjected to real-time PCR analysis with ApoB as the invariant control (the raw Ct number for ApoB was shown). For Table S2A, the mRNA level was calculated as the amount relative to that in livers of nonfasted control mice, which was arbitrarily defined as 1.0. For Table S2B, the mRNA level was calculated as the amount relative to that in livers of fasted control mice. Each value represents mean ± SEM of data from 4 mice group. *$p<0.05$ and $^{\#}p<0.01$ denote the level of statistical significance (two-tailed Student's t test) between control and *L-Chrebp*$^{-/-}$ mice under the same dietary condition. ChREBP-total real-time PCR primer was designed to amplify a C-terminal terminal region (absent in *L-Chrebp*$^{-/-}$ mice) shared by wild-type ChREBP-α and ChREBP-β. ChREBP-α and -β primers detect both wild-type and aberrant ChREBP-α and -β isoforms.

Supplemental Table S3. Relative gene expression in livers of sucrose-refed control and *L-Chrebp*$^{-/-}$ mice treated with AAV-GFP or AAV-nSREBP-1c

	Control		*L-Chrebp*$^{-/-}$	
AAV-	GFP	GFP	GFP	nBP-1c
	Fasted	Refed	Refed	Refed
**Phenotypic Parameters**				
Number of mice	5	6	4	5
Body weight (g)	24.7 ± 1.0	22.1 ± 0.4	21.2 ± 0.5	20.2 ± 0.8
Liver glycogen content (mg/g)	2.0 ± 0.4	68 ± 7	78 ± 0.2	82 ± 7
Liver cholesterol content (mg/g)	2.4 ± 0.1	1.6 ± 0.1	1.4 ± 0.1*	2.0 ± 0.1*
Liver triglyceride content (mg/g)	86 ± 9	6.3 ± 0.7	8.5 ± 1.5	37 ± 4*§
Plasma cholesterol (mg/dL)	129 ± 1	79 ± 2	60 ± 2*	59 ± 7*
Plasma triglyceride (mg/dL)	118 ± 6	118 ± 8	79 ± 9*	76 ± 10*
**Liver mRNAs**				
ApoB raw Ct number	17.2 ± 0.1	17.7 ± 0.3	17.5 ± 0.3	17.7 ± 0.2
**ChREBP and glucose metabolism**				
ChREBP-α	1.0	3.0 ± 0.3	1.1 ± 0.0*	1.3 ± 0.0*§
ChREBP-β	1.0	14 ± 3	0.1 ± 0.0*	0.2 ± 0.0*
L-Pyruvate kinase	1.0	21 ± 4.7	0.8 ± 0.1*	0.9 ± 0.1*
Ketohexokinase	1.0	7.1 ± 1.6	1.1 ± 0.1*	1.1 ± 0.1*
Triose kinase	1.0	10 ± 3	1.0 ± 0.2*	0.8 ± 0.1*
**SREBP pathway**				
SREBP-1a	1.0	4.9 ± 0.8	3.5 ± 0.3	3.0 ± 0.1
SREBP-1c	1.0	62 ± 9	24 ± 3*	27 ± 2*
SREBP-2	1.0	3.0 ± 0.5	4.6 ± 0.6	3.9 ± 0.3
**Fatty acid synthesis**				
ATP citrate lyase	1.0	85 ± 26	13 ± 2.2*	34 ± 4§
Acetyl-CoA carboxylase 1	1.0	23 ± 7	3.2 ± 0.3*	4.4 ± 0.5*
Acetyl-CoA carboxylase 2	1.0	5.0 ± 1.3	0.9 ± 0.2*	4.4 ± 0.5§
Fatty acid synthase	1.0	78 ± 21	9.2 ± 2.2*	14 ± 1*
Long chain fatty acyl elongase 6	1.0	36 ± 9	1.9 ± 0.2*	2.9 ± 0.3*
Stearoyl-CoA desaturase-1	1.0	3.9 ± 1.0	0.7 ± 0.1*	3.1 ± 0.4§
Glycerol-3-phosphate acyltransferase	1.0	11 ± 4	2.6 ± 0.5*	6.4 ± 0.8§
Malic enzyme	1.0	12 ± 4	0.8 ± 0.1*	2.5 ± 0.4*§
Glucose-6-phosphate dehydrogenase	1.0	7.4 ± 2.4	1.8 ± 0.1*	19 ± 3*§
6-phosphogluconate dehydrogenase	1.0	16 ± 5	3.5 ± 0.4*	4.9 ± 0.3*
**Cholesterol synthesis**				
HMG-CoA synthase	1.0	12 ± 2	22 ± 3*	13 ± 1
HMG-CoA reductase	1.0	15 ± 4	21 ± 4	18 ± 1

Littermate control and *L-Chrebp*$^{-/-}$ mice (male, two months of age) were injected via the tail vein with recombinant AAV-GFP (GFP) or AAV-nSREBP-1c (nBP-1c) (2x10^{11} gene copies per mouse). Seven days after the injection, the mice were fasted for 12 h or fasted for 12 h and then refed a 60% (w/w) high-sucrose/fat-free diet for 12 h prior to study. Total RNA was isolated from livers and subjected to real-time PCR analysis with ApoB as the invariant control. The mRNA level was calculated as the

amount relative to that in livers of fasted control mice, which was arbitrarily defined as 1.0. Each value represents mean ± SEM of data from 4-6 mice per group. Of note, selected RNAs from this experiment are also depicted in Fig. 6 but are normalized such that values from refed control mice are set to 1.0. *$p<0.05$ denotes the level of statistical significance (two-tailed Student's t test) between refed control (GFP injected) and *L-Chrebp*$^{-/-}$ (GFP or nBP-1c-injected) mice. §$p<0.01$ denote the level of statistical significance between sucrose-refed *L-Chrebp*$^{-/-}$ mice injected with GFP or nBP-1c. ChREBP-α and -β primers detect both wild-type and aberrant ChREBP-α and -β isoforms.
